# Distributed Resources Allocation Method for Space–Ground Integrated Mobile Communication System

**DOI:** 10.3390/s24144711

**Published:** 2024-07-20

**Authors:** Tingyin Zhao, Zhidu Li

**Affiliations:** 1 School of Communications and Information Engineering, Chongqing University of Posts and Telecommunications, Chongqing 400065, China; 2021210213@stu.cqupt.edu.cn; 2 Information and Communications Institute, Chongqing University of Posts and Telecommunications, Chongqing 400065, China

**Keywords:** space–ground integrated system, resources allocation, network slicing, GNSS

## Abstract

This paper presents an innovative approach towards space–ground integrated communication systems by combining terrestrial cellular networks, UAV networks, and satellite networks, leveraging advanced slicing technology. The proposed architecture addresses the challenges posed by future user surges and aims to reduce network overhead effectively. Central to our approach is the introduction of a marginal mobile station (MS)-assisted network resource allocation decision architecture. Building upon this foundation, we introduce the DP-DQN model, an enhanced decision-making algorithm tailored for MSs in dynamic network environments. Furthermore, this study introduces a feedback mechanism to ensure the accuracy and adaptability of the marginalization model over time. Through extensive simulations and experimental validations, our DP-DQN-based edge decision method demonstrates substantial potential in alleviating core network overhead while improving success access ratios compared to conventional methods.

## 1. Introduction

The integrated space–ground communication systems hold a promising future in the evolving landscape of mobile communication networks, particularly with their potential to expand coverage significantly [1,2,3]. Such systems are pivotal in advancing the Internet of Things (IoT), enabling a vast network of interconnected devices across more extensive and varied terrains [4,5,6]. Moreover, the fusion of these integrated systems with existing cellular networks promises to diversify and enrich the service types available to individual users, marking a significant leap forward in communication technology [7,8].

To realize the full potential of space–ground integrated communication systems, one crucial challenge is the efficient allocation of resources [9,10,11]. This involves the development of network slicing strategies and cross-network resource scheduling algorithms that take the unique characteristics of both satellite and cellular networks into account. Specifically, the location of MS will no longer be limited to the ground, but will expand and include sea and air [12,13,14,15]. Thus, certain devices can access not only the cellular network but also the satellite or drone network at the same time. When necessary, the MS also needs to switch between the network. Therefore, for the new generation of network resource allocation algorithms, it is necessary to include location information in order to maximize network efficiency and ensure seamless service delivery across the different layers of the integrated system.

Despite numerous advancements, current resource allocation methods, particularly those based on deep reinforcement learning techniques such as Deep Q-Networks (DQN) and Deep Deterministic Policy Gradient (DDPG) exhibit limitations. These algorithms often fall short in real-time performance and fail to consider the integration of multiple physical channels within the space–ground system, thus leaving considerable room for improvement in network efficiency. These gaps in existing methodologies underscore the necessity for our research.

To address these shortcomings, we introduce a novel integrated network slicing approach coupled with a Distributed Deployment Deep Q-Network (DP-DQN) resource allocation method. Our approach decentralizes the decision-making process by distributing the decision models to the user end, thus facilitating on-the-spot network resource decisions. This not only ensures real-time responsiveness but also reduces network overhead while accommodating the demands of various physical channels [16,17,18,19]. Through this innovation, our study seeks to significantly enhance the operational efficiency of space–ground integrated communication systems.

The contributions of this paper are summarized as follows:

- Firstly, we propose a new converged system based on the existing infrastructure of cellular and satellite communication networks, in which all resources are deployed in a unified manner rather than “separate and conquer”.
- Secondly, based on the idea of SDN, we propose a new network controller based on a distributed resource allocation decision-making model, which assigns the implementation body of decision-making behavior from the original core network controller to the access mobile terminal, so as to alleviate the dilemma of the rapid increase in access volume which will cause a huge burden on the core network in the future.
- Thirdly, based on DQN and negative feedback network technology, we propose a new DP-DQN model based on reinforcement learning for the overall system resource allocation, so that the mobile station can consider the problem of resource allocation from the perspective of the core network overhead, so as to make access requests conducive to the efficiency of the core network.

The remainder of this article is organized in the following order. The related works involved in this paper are reviewed in Section 2. In Section 3, the allocation system model is proposed and the optimization problem is formulated. Data rate optimization is investigated in Section 4 while physical network optimization is studied in Section 5. Finally, simulation and numerical results are presented and discussed in Section 6 followed by the Section 7.

## 2. Related Work

In this section, the paper introduces the related works from three aspects: network slicing, satellite navigation and resource allocation methods. In previous studies, these three topics were usually studied separately.

### 2.1. Network Slicing

Slicing technology is proposed to enhance the differentiated service capability of the network, which is mainly carried by the cellular mobile communication network. In recent years, the concept of satellite slicing has been proposed, so we introduce it from the following two parts: cellular network slicing and satellite network slicing.

InCellularScenarios, emphasis is usually placed on improving slice efficiency and increasing service types while reducing inter-slice interference. In [20], the author introduced a novel cooperative multi-agent reinforcement learning (RL) algorithm for RAN slicing, designed to adapt to variable slice numbers and effectively scale as they grow. In [21], the author formulated the slice-based service function chain embedding (SBSFCE) problem as an integer linear programming (ILP) that aims to fulfill differentiated requirements of flows. In [21], the proposed architecture leveraged new SDN extended federation modules in compliance with the ETSI requirements for inter-MEC system coordination. In [22], the author presented an innovative actor–critic Reinforcement Learning (RL) model named Slice Isolation based on RL (SIRL) to ensure the isolation between different slices while maximizing the user requests. In [23], the author proposed a QoS and security-oriented slicing resource allocation scheme in a multi-cell and multi-slice scenario in order to minimize the slice interference.

InSatelliteScenarios, emphasis is usually placed on improving resource usage efficiency and communication coverage. In [24], the author proposed a trust-based satellite Internet resource-slicing access authentication scheme to solve the efficient and secure access requirements in satellite communication. In [25], the author proposed a two-layer dynamic reconfigurable RAN slicing architecture for the ultra-dense low earth orbit satellite network (UD-LSN) to improve the slices’ efficiency. In [26], an architecture of IoT supportable satellite edge computing (SatEC) enabled by LEO satellite was proposed for global coverage extension and 3-D mobility enhancement. In [27], the author proposed an optimization satellite slicing framework able to exploit the available resources allocated to the defined network slices in order to meet the diverse QoS/QoE requirements exposed by the network actors. In [28], a hierarchical resource slicing framework was proposed for dynamic allocation of multidimensional resources to solve the problem of resource slicing and scheduling of joint 3C resources in a RAN edge scenario assisted by LEO content caching.

In summary, previous research on network slicing technology has regarded satellite and cellular networks as two independent individuals, and ignored the possibility of combining the two systems. This circumstance in the future wide coverage and diversified services of the new generation of mobile communication scenarios will lead to the problem of resource utilization.

### 2.2. Satellite Navigation

The Global Navigation Satellite System (GNSS) plays a pivotal role in delivering positioning services that are essential for a wide range of applications, from personal navigation to complex industrial operations. In recent years, there have been two research hotspots: satellite signal processing methods and navigation algorithms in severe climate situations.

InSignalProcessing, the focus of research is often on how to improve the positioning accuracy by changing the transmission sequence or modulation mode. In [29], the author proposed an optimization-based tightly-coupled precise point positioning (PPP) inertial navigation system (INS) vision integration method to achieve precise and continuous state estimation. In [30], the author presented a new broadband multi-carrier navigation modulation, namely orthogonal frequency division multiplexing with binary offset carriers (OFDM-BOC) in order to improve spectrum efficiency, tracking performance and anti-interference ability. In [31], a novel M-estimation-based robust iterated cubature Kalman filter (ICKF) was developed to minimize the impact of GNSS outliers while improving the correction effect of high-quality line-of-sight (LOS).

InSevereClimateAlgorithms, the focus of research is the algorithm design to reduce the interference in remote areas with severe climate. In [32], the author implemented the tightly coupled integration of navigation satellites with a low-cost micro-electromechanical system’s (MEMS) inertial measurement unit (IMU) to improve vertical accuracy and applied nonholonomic constraints to improve the standalone MEMS inertial navigation system (INS) performance during an outage. In [33], the author developed an adaptive–robust fusion strategy for low-cost GNSS systems which can provide reliable fusion positioning solutions when the GNSS signal is challenged.

In summary, navigation systems used to be studied as separate modules. Other navigation-related services simply invoke the navigation module as clients. Such a fragmented design approach obviously has a large room for improvement in resource utilization and system reliability, especially in many emerging scenarios that require navigation system assistance.

### 2.3. Network Decision Method

In the research on network resource allocation, there are two main aspects: traditional non-AI resource allocation algorithms and AI-assisted intelligent resource allocation algorithms. Because of its unique flexibility, the latter can often greatly improve the efficiency of the network.

Innon-AIMethods, a distributed power allocation was performed in [34] to optimize the performance of cell-edge users by Lagrange method. In [35], the author introduced a distributed structure for the resource allocation problem by forming a convex optimization problem, maximizing the overall system utility function. In [36], a new resource allocation optimization and network management framework for wireless networks using neighborhood-based optimization has been proposed instead of fully centralized or fully decentralized methods to reduce interference and increase capacity.

InAI-basedMethods, researchers are aiming to offer a more dynamic and responsive approach to resource allocation. In [37], the author proposed a multi-agent deep reinforcement learning (DRL) approach with an action space reduction strategy to achieve the dynamic VNF orchestration, backup and mapping solution. In [38], the author proposed a resource allocation (RA) method using dueling double deep Q-network reinforcement learning (RL) with low-dimensional fingerprints and soft-update architecture (D3QN-LS) based on a Manhattan grid layout urban virtual environment. In [39], an improved deep Q-network (DQN) algorithm was introduced to improve the efficiency of resource utilization. In [40], the author investigated the dynamic offloading of packets with finite block length (FBL) in an edge-cloud collaboration system consisting of multi-mobile IoT devices (MIDs) with energy harvesting (EH), multi-edge servers and one cloud server (CS) in a dynamic environment based on a multi-device hybrid decision-based DRL solution. In [41], based on the enhanced K-mean algorithm and multi-agent PPO (MAPPO) algorithm, a cooperative trajectory design method was proposed for the UAVs to minimize interaction overhead and optimize deployment efficiency. In [42], the author proposed a novel algorithm based on Soft Actor–Critic (SAC) to solve the system cost minimization problem considering vehicle users’ satisfaction, RSUs’ cost and vehicle workers’ reward.

In summary, although AI-based network resource allocation methods solve the problems of traditional non-AI methods in terms of flexibility, the current AI methods, especially the use of deep network cases, still have problems when talking about real-time processing. In addition, these methods have rarely taken space–ground integrated circumstances into consideration.

### 2.4. Motivation of Our Works

In summary, current research on network slicing, satellite navigation and resource allocation is poor in terms of the converged control and real-time requirement emphasized by the integrated space–ground communication system, which motivates this paper.

## 3. System Model

In this section, we consider a new kind of distributed resources allocation structure based on the slicing technology of the space–ground integrated communication system.

### 3.1. Network Scenario

**Figure 1:**
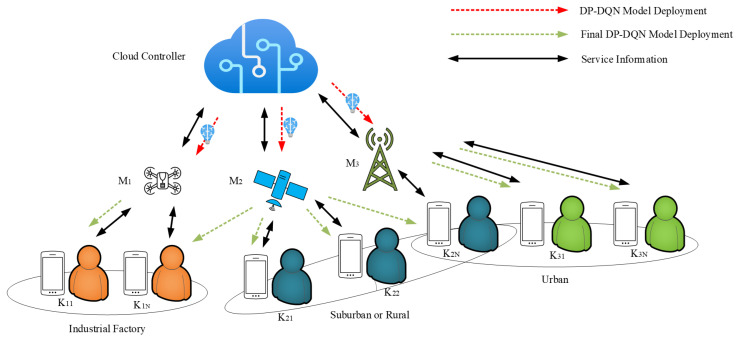
The deployment of decision model: the decision model will be pre-trained on the core network controller and sent to the MS.

In the architecture designed by us, there are three types of physical information carriers, namely satellite networks, cellular networks and drone networks, which are marked as $M_{1}$, $M_{2}$ and $M_{3}$. At the same time, each physical channel contains three network slices, $T_{1}$, $T_{2}$ and $T_{3}$. By the way, we define a virtual concept as a ‘location area’. As shown in Figure 1, each ‘area’ contains several MSs with the similar properties.

Also, each of the networks $M_{i}$ will serve a number of MSs. For example, the distances between users from $K_{21}$ to $K_{2N}$ and certain base stations are relative high, where we call them sub-urban or rural areas. In these circumstances, we introduce a satellite network that acts as the carrier of eMBB service instead of cellular network.

In Figure 1, we can see a special access device, the $K_{2N}$. For the same kind of service slice, its state is bounded between suburban and urban areas, so both networks can be used as its carrier.

In order to select a more suitable network for access, we conduct our works based on core network overhead, which means the network access decision will be made by the MS from the perspective of network overhead.

The whole decision process of network access at the MS side will follow the procedure below.

- First of all, the MS will analyze the data size and service type of the data to be sent, and determine its transmission power.
- Secondly, this information is fed into the DP-DQN model (discussed in detail later in this article) delivered from the network side and determines the type of network that is ultimately accessed.

Different from the traditional network, the training and deployment process of DP-DQN model is based on SDN (Software Defined Network). Here, we introduce the DP-DQN deployment method.

**Figure 2:**
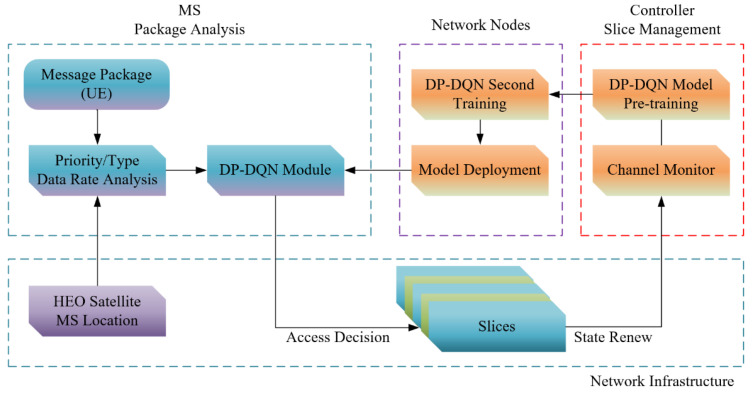
Distributed network decision-making structure.

Firstly, as shown in Figure 1 and Figure 2, the basic model will be trained at the side of the core network controller. Specifically, the input parameters are service types, average data volume and current network congestion. After this process, the pre-trained model is sent to each network carrier without distinction. The red dashed line in Figure 1 represents the model transfer flow for this process.

Secondly, each network will then train the model twice, adding its own parameters based on the model of core network controller, such as the signal transmission power on the network side, the average delay of the network and the user amount in the coverage.

Finally, these secondary training models will be passed to the MS at the edge, where these models will undergo the final training. Here, the third (final) training process at the MS side will also conduct integrated learning with all the models received. Here, the training of the model will take the following parameters into account: the user’s own average amount of data, location information (i.e., latitude and longitude altitude), the state of the three networks at this time and the records of past resource decisions. Thus, what the core network controller should do is to keep monitoring the crowd state while renewing the basic decision model.

The parameters included in the three training sessions are summarized in Table 1.

**Figure 3:**
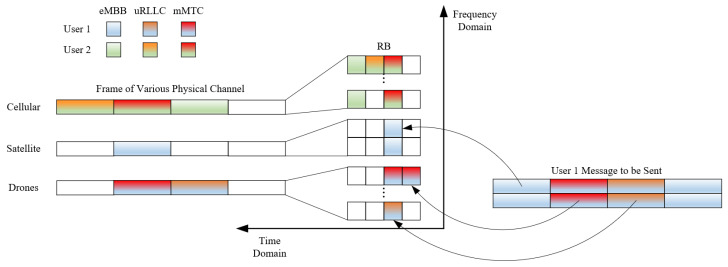
The diagram contains three service types (three slices) for two users. Here, user 1 is located in countryside industrial areas away from the cellular network, so the eMBB service is mapped to the satellite network, while mMTC is mapped to the drone network.

The overall frequency resource mapping relationship is shown in Figure 3. On the left is the frame structure of the three physical networks, in the middle is the time–frequency domain resource allocation diagram and on the right is the data packet to be sent by the user. The data to be sent by the user are first analyzed to determine the type of service they belong to. The data are then mapped to the appropriate resource block. Finally, the access network is determined according to the user’s location and other related information.

In Figure 2, the allocation decision-making process is given, consisting of three aspects, package and access strategy analysis at the MS side, slice management at the side of the core network controller and the location analysis provided by navigation systems (GNSS).

Figure 2 is consistent with what is described in the previous chapters, and we further elaborate it from the perspective of model training. In the initial training stage, the DP-DQN model will first be trained based on the reinforcement learning method, and then in the process of continuous iterative update of the initial model, data from Channel Monitor will be used to improve the adaptability of the model. Then, the secondary training will be performed on each network node before the final deployment of the model. On the client side, the data to be sent are packaged by Message Package block, analyzed in the Type Analysis module and given a priority. At the same time, the current MS location information is obtained by the MS location module. Each time data are sent, the above information is obtained, and combined with the final resource allocation result and system feedback, the MS side will form a data pool. Subsequent edge DP-DQN models will be trained based on this.

### 3.2. User Request Procedure

To illustrate the network access process, we take one MS ($K_{2N}$) as an example, using the architecture in Figure 1 and Figure 2.

Firstly, the core network controller sends the pre-trained model with the real-time crowd state to all the physical network carriers, and then these nodes send the model to the $K_{2N}$ after secondary training.

Secondly, $K_{2N}$ in the certain area $M_{2}$ ask for a connection to send messages through the network. Here, the message will be analyzed by the designed algorithm to give it a classification of service type and allocated data rate at the same time. Also, $K_{2N}$ gets its location (i.e., longitude, dimension and altitude) from the HEO navigation satellite as other parameters.

Thirdly, the algorithm embedded in MS will use these parameters to calculate the most suitable RB requirement with transmitting power. Then, $K_{2N}$ will employ the DP-DQN model to decide which physical network (e.g., Satellite Network eMBB Slice) to switch in. Here, for $K_{2N}$ which is located in the near-urban area, the slices allocated from the satellite will be much more expensive than those from the cellular network.

Finally, the analysis result (service type, access network and needed RB number) will be sent to a particular network. Available slice blocks will then be allocated to the user to meet the demand of data rate. If the current network no longer has enough resources due to resource requests from other users, the core network will meet its needs as far as possible according to the principle of proximity. If this is still not satisfied (in practice, such a scenario is very rare), feedback will be sent to the mobile station to recalculate the appropriate access network.

### 3.3. Network Update Process

In our design, the update of the network will consist of two parts: the core network controller and the MS controller. The former is mainly to update the current usage and congestion index of each slice and network in real time, while the latter is to update the location of MS in real time.

#### 3.3.1. Channel State Update

The channel state monitor block will keep updating every *t* minutes, checking the usage rate of each slice.

#### 3.3.2. Positioning Update

The location monitor block will keep asking for new data from the HEO satellite every *t* minutes.

The parameter *t* is not concrete which will change depending on the characteristics (e.g., speed or altitude) of the MS.

Since the whole process and decisions are made by the MS, there may be some mistakes due to the message latency. For example, when MS performs slice selection analysis, because the network has not had time to update the latest network situation for MS, the slice selected by MS may be assigned to other users at this time, but MS does not know this situation, resulting in allocation (access) failure.

**Figure 4:**
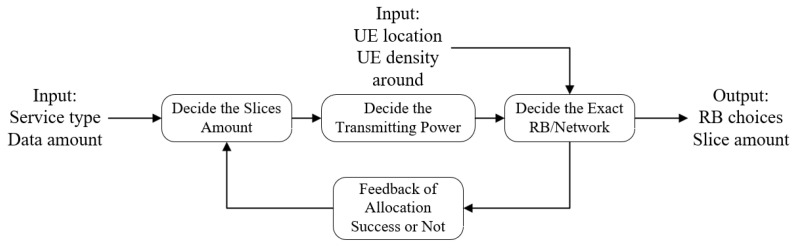
Distributed network decision-making process flow.

Therefore, the proposed procedure contains a feedback loop which is designed to solve the possible volatility of the network, as you can see in Figure 4. Every failure record of allocation will be transmitted backward to the first step to lower the fail rate of the next allocation decision.

### 3.4. Problem Formulation

As is mentioned above, to relieve the core network overhead, we shift some of the work of allocation from the original core network controller to the mobile station side, that is, the mobile station will first determine which physical slice (satellite or cellular network) is the most suitable for itself. Considering that slices are different in the latency and the channel atmosphere, we introduce a parameter C to describe the cost of certain slices to help the MS make the decision.

(1) $$C=\left[\mathrm{Cost}\right]=G\left(T,\;R^{\prime },A\left(t\right),P_{kb}\left(t\right)\;\right)$$

(2) $$=\lambda_{1}T+\lambda_{2}R^{\prime }+\lambda_{3}A\left(t\right)+\lambda_{4}P_{kb}\left(t\right)$$

where $R^{\prime }$ denotes the actual allocated data rate of users

(3) $$R^{\prime }=RG_{e}\left(R,T\right)=R\frac{G(R,T)}{100}$$

among which, $G_{ij}\left(R,T\right)\in \left[0,100\right]$ is an urgency factor to describe how much content in the package is valuable to be given the first priority, which has been fully investigated in [43,44,45].

The resources allocated for user access should be as small as possible under the premise of meeting its priority and data volume, so as to improve the data utilization efficiency of the system. Hence, the allocation procedure can be transformed to an optimization problem.

(4) $$\underset{A\left(t\right),P_{kb}\left(t\right),R^{\prime }}{\mathrm{minimize}\;}\left(C\right)=\underset{A(t)}{\mathrm{minimize}}\left(G\left(T,R^{\prime },A\left(t\right),P_{kb}\left(t\right)\right)\right)$$

(5) $$s.t.\;\;A_{ij}\left(t\right)\in \left[0,1\right],1\leq i\leq 3,1\leq j\leq N$$

(6) $$\sum_{i=1}^{3}\sum_{j=1}^{N}A_{ij}\leq C_{ch}$$

Here, in our research, we propose three types of slices as mentioned before, thus, $T=Z^{+},\left[1,3\right]$ which is a 1×N array where N indicates the number of total requests. Parameter $A=R^{+},\left[0,1\right]$ is a 3×N array aimed to describe the state of every slice and RB (used, crowed, etc.) which is positively correlated with channel allocation cost in the formula. P indicates the density of MS around a particular mobile devices.

Sorting through the optimization problems we just listed, we can get the following formula.

(7) $$P1:\underset{A\left(t\right),P_{kb}\left(t\right),R^{\prime }}{\mathrm{minimize}}\left(\lambda_{1}T+\lambda_{2}R^{\prime }+\lambda_{3}A\left(t\right)+\lambda_{4}P_{kb}\left(t\right)\right)$$

(8) $$s.t.\;\;\sum_{i=1}^{3}\sum_{j=1}^{N}A_{ij}\leq C_{ch}$$

(9) $$T_{ij}\in \left\{1,2,3\right\},1\leq i\leq 3,\;1\leq j\leq N$$

(10) $$A_{ij}\left(t\right)\in \left[0,1\right],1\leq i\leq 3,1\leq j\leq N$$

The problem is to find the best couple A,P and $R^{\prime }$ to minimize the cost function. Formula (7) restricts the maximum capacity of the logical channel, while formula (10) defines the allocation state of the slice. Corresponding to the channel slicing problem, the goal of each MS when asking for access to the network is to find the best physical network to switch in reflected by A, the transmitting power P and proposed data rate $R^{\prime }$.

However, the optimization problem listed here is a NP-hard problem which cannot be worked out by the MS quickly. Thus, we divide the whole problem into two sub-optimization problems: allocated data rate optimization, P2, and allocated physical network optimization, P3.

The first optimization problem P2 is to find the optimal transmit power and the number of resource blocks to meet the needs of users under the assumption of a physical distribution network. In the previous section, we discussed the goal for the MS in the resource allocation problem P1 as a minimization problem, where one of the goals is to minimize $R^{\prime }$. In other words, it is to make *R* as large as possible, but not more than $R^{\prime }$, which is the minimum transmission rate to meet the requirements of k service.

(11) $$P2:\underset{P_{kb}\left(t\right),Ø}{\mathrm{maximize}\;}\left(R_{k}\right)=\sum_{b\in B}ØB_{kb}\frac{\sum_{b\in B}a_{kb}\left(t\right)}{M}\times \log_{2}\left(1+\frac{P_{kb}\left(t\right)h_{kb}\left(t\right)}{\sigma_{k}^{2}\left(t\right)}\right)$$

(12) $$s.t.\;\;R_{k}\leq R_{k}^{\prime }$$

(13) $$\sum_{b\in B}B_{kb}\leq B_{b}$$

(14) $$\sum_{b\in B}P_{kb}\leq P_{b}$$

(15) $$\sum_{i=1}^{3}\sum_{j=1}^{N}A_{ij}\left(t\right)\leq C_{ch}$$

(16) $$T_{ij}\in \left\{1,2,3\right\},1\leq i\leq 3,\;1\leq j\leq N$$

(17) $$A_{ij}\left(t\right)\in \left[0,1\right],1\leq i\leq 3,1\leq j\leq N$$

In the above optimization problem, the symbol χ represents the proportion of resource panes that can actually be used in an allocated resource block.

(18) $$\chi =\frac{\sum_{b\in B}b_{kb}\left(t\right)}{M}$$

The parameter $b_{kb}\left(t\right)$ describe the state of each slice (i.e., whether it will be allocated to current service T or not) which is a crucial factor in the allocation process.

(19) $$b_{kb}\left(t\right)=\left\{\begin{array}{cc} 1, & \mathrm{if}\;the\;slice\;has\;been\;allocated\\ 0, & \mathrm{else} \end{array}\right.$$

The second optimization problem is to select the most appropriate allocated physical channel according to the characteristics of the users, so as to minimize the total overhead when the transmission power and the number of allocated resource blocks are determined.

(20) $$P3:\underset{A(t)}{\mathrm{minimize}\;}\left(C\right)=\underset{A(t)}{\mathrm{minimize}}\left(G\left(T,R^{\prime },A\left(t\right),P_{kb}\left(t\right)\right)\right)$$

(21) $$s.t.\;\;A_{ij}\left(t\right)\in \left[0,1\right],1\leq i\leq 3,1\leq j\leq N$$

(22) $$\sum_{i=1}^{3}\sum_{j=1}^{N}A_{ij}\leq C_{ch}$$

The sub problems P2 and P3 will be discussed in the following sections. The whole solving process has been illustrated in Figure 4. All the variables defined in this article are summarized in Table 2.

## 4. Data Rate Optimization

In our research, logical channels are defined from the perspective of the system, that is, a logical channel contains different mappings of physical channels, so different resource blocks correspond to different channel noise and raw resource allocation. Therefore, when solving the optimization problem, the MS needs to solve *R* separately in several different physical mappings and get the final optimal scheme.

Thus, the problem P2 can be conducted in these two process named as:

- P4: First, it is assumed that in all physical mapping cases, the transmitted power of MS is the same. On this basis, R is calculated and the optimal allocation resource block amount is selected.
- P5: Then, when the allocated resource block is determined, the transmission power $P_{kb}\left(t\right)$ is changed to obtain the optimal transmitting rate R.

### 4.1. P4 Optimization

For a given transmitting power and the current slice state, the RB allocation question can be formulated as

(23) $$P4:\underset{Ø}{\mathrm{maximize}\;}\left(R_{k}\right)=\sum_{b\in B}ØB_{kb}\frac{\sum_{b\in B}a_{kb}\left(t\right)}{M}\times \log_{2}\left(1+\frac{Ph_{kb}\left(t\right)}{\sigma_{k}^{2}\left(t\right)}\right)$$

(24) $$s.t.\;\;R_{k}\leq R_{k}^{\prime }$$

(25) $$\sum_{b\in B}B_{kb}\leq B_{b}$$

(26) $$\sum_{i=1}^{3}\sum_{j=1}^{N}A_{ij}\left(t\right)\leq C_{ch}$$

(27) $$T_{ij}\in \left\{1,2,3\right\},1\leq i\leq 3,\;1\leq j\leq N$$

(28) $$A_{ij}\left(t\right)\in \left[0,1\right],1\leq i\leq 3,1\leq j\leq N$$

where Ø is a matrix of $\chi_{kb}$.

We design an optimization Algorithm 1 for the MS to find out the proper slices named FPCB (Fixed Power Change the Block).
**Algorithm 1** Algorithm of FPCB (P4)**Require:** *A*, *P*, $B_{kb}$, *P* (This is Inputs)**Ensure:** *B*, *A* (This is Outputs)1:Set the initiate solution $B^{\left(0\right)},P,A$2:**while** *A* is not full **do**3:   calculate the capacity of each RB4:   **for** each n∈[1,length(A)] **do**5:     **if** $A_{n}$ is not full **then**6:        **for** each $i\in [1,length\left(A_{n}\right)]$ **do**7:          allocate this slice to the MS8:          calculate the rate of this slice in the block n9:          *R* = *R* + $R_{i}$10:        **end for**11:     **else**12:        jump to the next RB13:     **end if**14:     Compare which of the block has a higher rate and select it as *B*15:     Try other kind of *B*16:     Renew the channel allocation *A*17:   **end for**18:**end while****return** Outputs *B*, *A*

### 4.2. P5 Optimization

Now that the proper slices of RB have been found out, the task is to change the transmitting power accordingly to get the result.

(29) $$P5:\underset{P_{kb}\left(t\right)}{\mathrm{maximize}\;}\left(R_{k}\right)=\sum_{b\in B}ØB_{kb}\frac{\sum_{b\in B}a_{kb}\left(t\right)}{M}\times \log_{2}\left(1+\frac{P_{kb}\left(t\right)h}{\sigma_{k}^{2}\left(t\right)}\right)$$

(30) $$s.t.\sum_{b\in B}P_{kb}\leq P_{b}$$

Since we assume that the data of the same service request from the same MS will all be mapped to the same type of physical channel (for example, language information from user A in remote areas can all be mapped to satellite channels), the channel matrix here can be set to be the same when the transmission power is optimized, that is, the transmission power of each resource block is the same. Thus, we change the restriction of the P3 as

(31) $$s.t.P_{kb}=\frac{P_{b}}{N}$$

## 5. Physical Network Optimization

In the previous section, the algorithm has given several feasible physical RBs for the MS to switch in. The goal of this section (the third step in Figure 4) is to propose a method to finally choose one particular network among the given choices based on the analysis of penalty function.

We propose a new DP-DQN algorithm based on deep reinforcement learning in this section to solve the optimization problem—select the appropriate access physical channel to minimize the overall network cost (i.e., from the perspective of core network overhead instead of single MS).

It is worth mentioning that the training method and use of DQN used in this study are slightly different from the conventional situation. In this study, the training of DQN is located in the controller side of the core network, while the actual deployment of the model is located in the MS side. This design is because we hope to achieve an equivalent feedback loop through core network controller training and marginal terminal deployment, so that the mobile terminal can consider the access selection problem from the perspective of core network overhead.

After the training is completed, DQN will be deployed to the mobile terminal. Here, we draw on the idea of federated learning by deploying the edge decision model while absorbing the real-time data of each mobile station for final training to improve the accuracy of the model and the overall efficiency of the network. So we call it DP-DQN, or Distributed-Deployed DQN.

**Figure 5:**
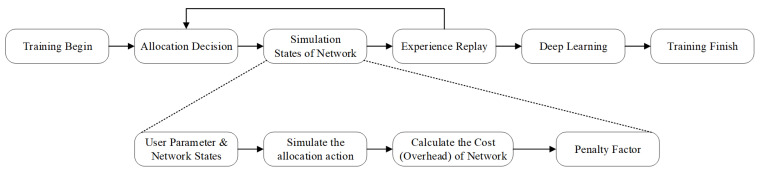
Proposed DP-DQN pre-training process at the side of the core network controller.

As you can see in Figure 5, during the training process, we will simulate some of the possible access request from the MS to the network. Then, the cost of certain actions will be calculated based on the designed formula to estimate the overhead of this allocation decision made on the network.

We define the actions State Space and Action Space here. Instead of the Reward Function in the previous section, we define a Penalty Function to put the most emphasis on the action of the MS. In other words, if the MS makes a request which will put more burden on the core network, penalty will be given to the MS to make sure the overhead of the network is being kept at a relatively low level.

### 5.1. State Space

The state space of the proposed model is the current crowd situation of the network which is sent in the form of periodic packages to the mobile devices. The definition of this part is similar to that of Q-learning. The difference is that here, we focus more on the overhead of the network as a state factor.

(32) S(t)=A(t),k∈K,b∈B

Here, in addition to slicing state information similar to traditional Q-learning and DQN, we introduce some real-time user state information in order to better allocate network resources (e.x. user location, transmitting power, user data amount, etc.). Thus, the state space can be summarized as follows:

(33) $$S\left(t\right)=\left[A(t),P_{kb}\left(t\right),D_{k}\left(t\right),R_{k}^{\prime }\left(t\right)\right],k\in K,b\in B$$

where $D_{k}\left(t\right)=\left\{L_{c}\left(t\right),L_{u}\left(t\right),\rho (t)\right\}$, which is the location status parameter of the mobile device and represents the shortest distance from the mobile station to the cellular and UAV network access point and MS density in certain areas, respectively, collected and analyzed by the satellite navigation module.

(34) $$L_{c,2,1}\left(t\right)=\sqrt{{d_{2,1}\left(t\right)}^{2}+{\left(h_{2}\left(t\right)-h_{1}\left(t\right)\right)}^{2}}$$

(35) $$\rho \left(t\right)=\;\frac{card(U(t))}{\pi R^{2}}$$

**Figure 6:**
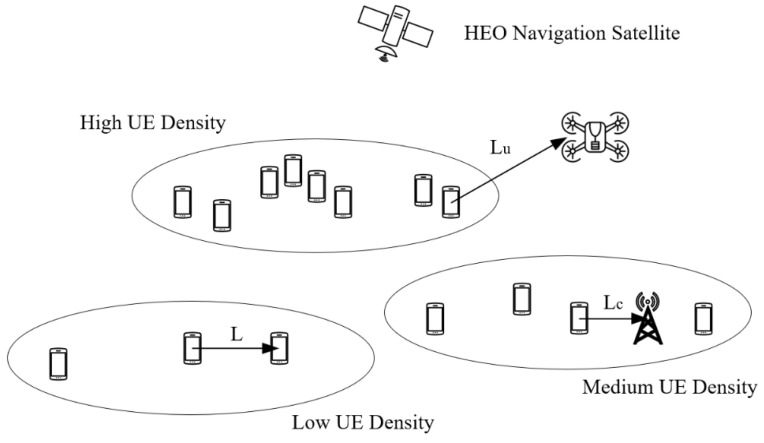
Schematic diagram of user density per unit area.

The parameters in the above two formulas can be obtained in the following ways. The position relationships between mobile stations are shown in Figure 6.

(36) $$d_{2,1}\left(t\right)=2rarcsin\sqrt{sin^{2}\frac{\Delta \phi_{2,1}}{2}+cos\phi_{1}cos\phi_{2}sin^{2}\frac{\Delta \theta_{2,1}}{2}}$$

(37) $$\Delta \phi_{2,1}=\phi_{2}-\phi_{1}$$

(38) $$\Delta \theta_{2,1}=\theta_{2}-\theta_{1}$$

(39) $$U\left(t\right)=\left\{\left(x,y\right)|\sqrt{{\left(x_{i}-x\right)}^{2}+{\left(y_{i}-y\right)}^{2}}<R\right\}$$

### 5.2. Action Space

The action space is defined as the allocation of slices for the new access mobile devices which can be divided into two steps:

- Give the slice choice $B_{kb}$ which indicates that the MS will select which specific slicing network to access, and also select several specific slicing resources in this network
  (40) $$a\left(t\right)=B_{kb},k\in K,b\in B.$$
- Renew the slice state from $A_{kb}\left(t\right)$ to $A_{kb}\left(t+\right)$.

For example, the current number of $A_{i,j}\left(t\right)$ is 0 which means it is available now; the action may allocate it to the new MS and set it to 1.

### 5.3. Penalty Function

The definition of a penalty function is to make the model trying best to achieve the highest efficiency at certain circumstance. In other word, the decision will be made by the MS from the perspective of releasing the overhead of core network.

In order to make the difference between the slices of different maps more obvious (e.x. satellite, cellular or low altitude drone network), we define the penalty function target-that is, we define the penalty function separately for the three types of networks.

- Penalty Function of Satellite Network
  (41) $$\begin{array}{c} {\mathrm{PW}}_{1}\left(t\right)=-log\left(G^{\prime }\left(\mathrm{DA},\;R^{\prime },L_{c},L_{u},P_{kb}\left(t\right)\;\right)\right)\\ =-log(\mathrm{DA}+\lambda_{11}*R^{\prime }+\lambda_{12}*L_{c}+\lambda_{13}*L_{u}+\lambda_{14}*P_{kb}\left(t\right)) \end{array}$$
- Penalty Function of Cellular Network
  (42) $$\begin{array}{c} {\mathrm{PW}}_{2}\left(t\right)=-log\left(G^{\prime }\left(\mathrm{DA},\;R^{\prime },L_{c},P_{kb}\left(t\right)\;\right)\right)\\ =-log(\mathrm{DA}+\lambda_{21}*R^{\prime }+\lambda_{22}*L_{c}+\lambda_{23}*P_{kb}\left(t\right)) \end{array}$$
- Penalty Function of UAV Network
  (43) $$\begin{array}{c} {\mathrm{PW}}_{3}\left(t\right)=-log\left(G^{\prime }\left(\mathrm{DA},\;R^{\prime },L_{u},P_{kb}\left(t\right)\;\right)\right)\\ =-log(\mathrm{DA}+\lambda_{31}*R^{\prime }+\lambda_{32}*L_{u}+\lambda_{33}*P_{kb}\left(t\right)) \end{array}$$

If we combine these three cases into one formula, we get the following more general case.

(44) $$\begin{array}{c} \mathrm{PW}\left(t\right)=-log\left(G^{\prime }\left(T,\;R^{\prime },S\left(t\right),P_{kb}\left(t\right)\;\right)\right)\\ =-log(\mathrm{DA}+\lambda_{1}*R^{\prime }+c_{1}*\lambda_{2}*L_{c}+c_{2}*\lambda_{3}*L_{u}+\lambda_{4}*P_{kb}\left(t\right)) \end{array}$$

Here, the parameter DA and P has been determined by the previous sections, while $\lambda_{i}\in \left[0,1\right]$ is a parameter aimed to describe the sensitiveness of the system. Moreover, $C_{i}\in \left\{0,1\right\}$ is the network label of the system, which is used to indicate the difference in the penalty function corresponding to different physical networks (i.e., cellular network or satellite network).

It is worth mentioning that in the process of training, the system dynamically adjusts the value of sensitive factors according to the current network state and other information. This is an optimization problem with only four finite variables, so it can be quickly solved by existing optimization algorithms or grid search methods.

## 6. Numerical Results

In this section, we build a simulation of the architecture we proposed in MATLAB (Version R2020a) and Python (Version 3.10) to experimentally simulate the real allocation decision circumstance at the MS side depending on the model deployed by the controller of the core network so as to prove the proposed better performance. The result will be given about the access ratio as well as the cost of the calculation resources of the devices in the form of the CPU usage.

### 6.1. Simulation Procedure

**Figure 7:**
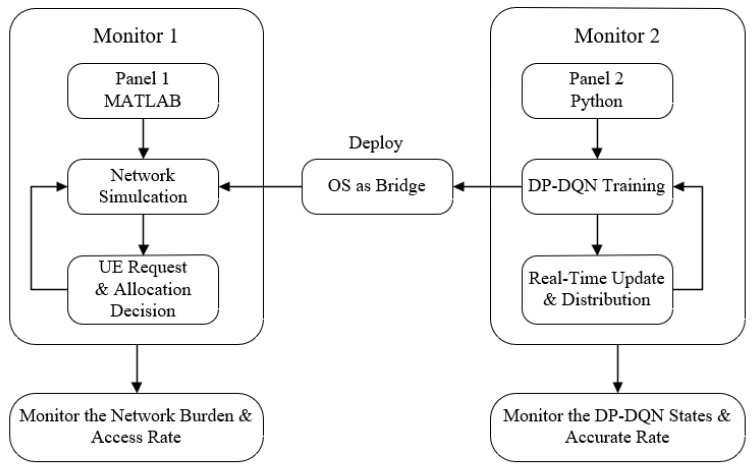
Simulation flow diagram: the DP-DQN model is trained in python and transferred to MATLAB with the operating system as a bridge to simulate the process of decision model delivery.

The architecture of the whole experimental approach to our ideas in this study is shown in Figure 7.

We first define MS objects programmatically in MATLAB, and randomly generate its arrival time, transmit power, packet size and other parameters for each object. Then, we use python to conduct the DP-DQN reinforcement learning training proposed by us. After obtaining the model, the operating system of PC is used as a bridge to pass the trained model into the MATLAB function. This step also simulates the distributed deployment process of the model trained by the core network to the user’s mobile station.

In the simulation, we assume that the maximum amount of users over a 1 h duration is 3000, among which 1500 users are asking for a connection to send messages while the other 1500 users are asking for slices to receive messages. The max information package of one request will not exceed 20 GB and will not be below 2 MB. At the same time, each message package will be given an emergence level randomly, which is a positive integer between 1 and 3. We will define three types of service types which have different data rates and latencies. Every user will have a reference service type. To make the simulation more universal, all of the parameters listed above will be given randomly in the simulation process. Meanwhile, the requests from the users have different arrival times, which will be randomly generated in the loop. And, in our research, we assume that the arrival time of each user obeys the Poisson process.

Also, to take the location and density of the MS, we will also arrange location (longitude, latitude and altitude) for each MS (i.e., user) randomly. To simulate the scenario of metropolises with rural areas. The generation of the location will not be absolutely random; most of the users’ locations will be around the main city which is set as 4 in our work and several users will be around the small towns, which means in these kind of area the access request will not be as much as those in metropolises.

In the table, the element SF indicates Sensitive Factor. Moreover, we will define three types of physical channels (e.g., terrain cellular, satellite and drone network). Each channel will be cut into three slices based on the total service types. Then, the slices will also contain 1800 or less different sub-carrier. The minimum particle have a maximum transmitting rate of 3 MBps. In addition, $\lambda_{i}$ in the table is the sensitive factor of each input parameter, which is set as variable (non-fixed value), and experiments are conducted on different $\lambda_{i}$. Here, we use a grid search algorithm with a step size of 0.1 to determine the most appropriate value in a certain state, shown in Table 3.

All of the slices which have been allocated to users will be recorded in an array until the process of transmitting messages has been finished.

### 6.2. Network Access Ratio Performance

As we mentioned in the previous chapters, we introduced the concept of simulation time in the design of the system simulation model, which is the generation of random numbers for the user request issue time. In other words, when the simulation duration is determined, changing the number of access users is equivalent to changing the user access request per unit time, that is, the number of user access densities in the time domain.

In order to better show the difference between decision-making methods, we selected four groups of data samples as comparative experiments towards the proposed method. Also, we set different SFs of the penalty function during the simulation, considering the existence of multiple parameters that may have different impacts on the system. Because there are not many combinations of SFs, they can be easily found by periodic grid search in practical systems. Here, we chose four typical sets of SFs for presentation summarized in Table 4.

**Figure 8:**
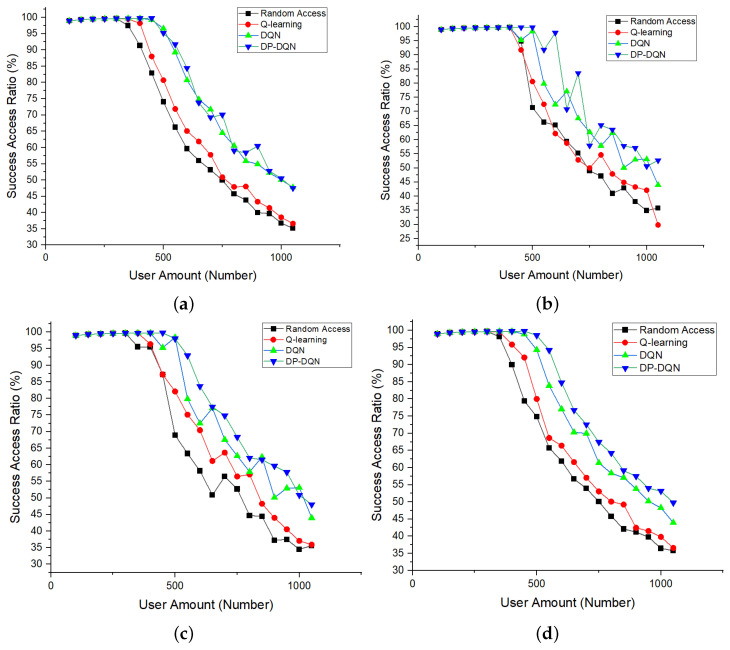
MS successful access ratio of proposed slicing method with different user amounts in the circumstances of different sensitive factors summarized in Table 4. Since the differences of sensitivity factors in (**a**–**c**) are relatively small, the impact of different parameters on the system cannot be reflected, so its effect is slightly lower than that of (**d**).

As shown in Figure 8, the method without any advanced resource allocation called Random Access here has the lowest access ratio. Also, the basic access resource allocation algorithm powered by Q-learning is shown, that is, the so-called “Channel Cost” method proposed by many scholars in the past. It can be clearly seen that our proposed DP-DQN method has great advantages over other methods, especially when the unit time access volume gradually increases. It is worth mentioning that in this group of experiments, while changing the number of users making requests per unit time, the number of resources available for deployment per unit time in the control network remains unchanged, and the arrival time of users is subject to the same random process (Poisson process).

**Figure 9:**
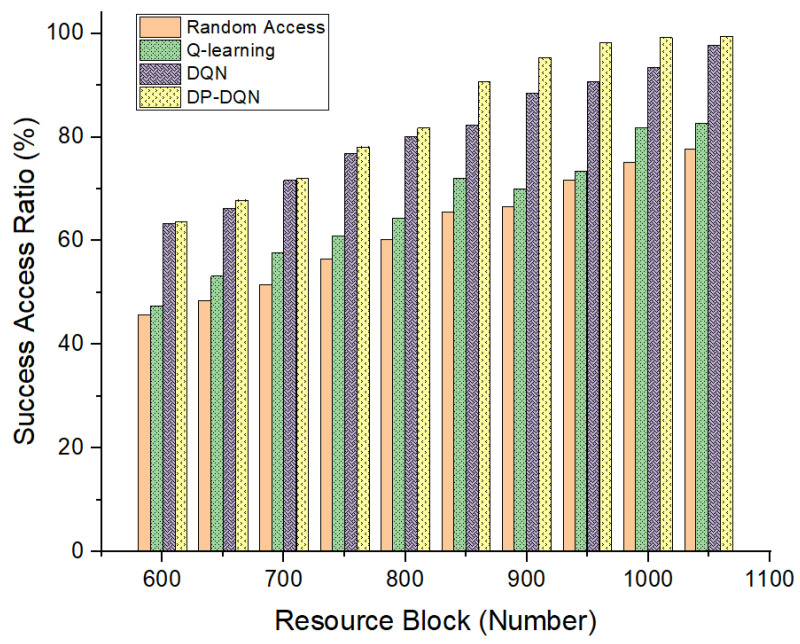
MS successful access ratio of proposed slicing allocation method with different RB amounts.

Secondly, we fixed the number of users unchanged, changed the number of resources in the frequency domain (i.e., the amount of total resource blocks) and adopted the SF pairs that worked best in the previous experiment. It can be seen that our proposed resource allocation method has a higher access ratio than the original methods, shown in Figure 9.

Compared with the experimental group, it is still consistent with the situation just now, and it can be seen that the method we proposed has obvious advantages in the number of resources from 600 to 1000. Especially when the number of resources is relatively short compared to the number of access users, our decision-making method can see a rapid increase in the access success rate, which shows that the method has excellent robustness in the face of sudden large access.

### 6.3. Network Overhead Performance

**Figure 10:**
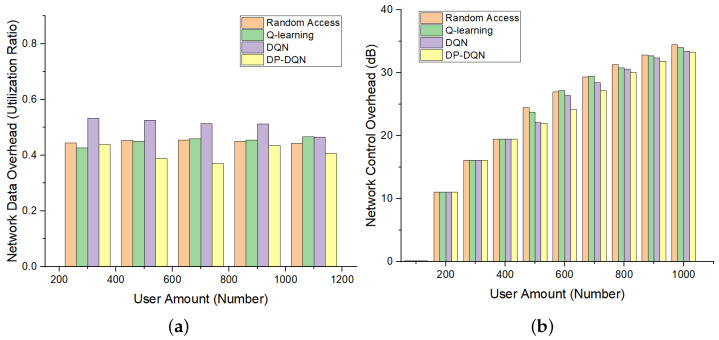
(**a**) User panel overhead of proposed slicing method with different user amounts. (**b**) Control panel overhead of proposed slicing method with different user amounts. In the figure, we compare our proposed approach to the current mainstream solution, which can better reduce the network overhead with the same amount of resources and users.

Finally, to further verify the superiority of our proposed method, we define a network overhead function in two aspects (i.e., user panel and control panel). Figure 10 shows the corresponding network overhead of these two panels for different user access quantities. It can be seen that with the same user access volume, the proposed method causes the least network overhead.

Network user panel overhead, that is, the burden brought by the data service carried in the network, which can be measured by the data flow in the network, as shown in the following formula.

(45) $$Bd_{u}=E(\frac{RB_{used}}{RB_{total}})=E(\frac{\sum_{k\in M}B_{kb}}{C_{ch}})$$

The control surface overhead is defined by the transmission volume of the signaling, as shown in the following formula.

(46) $$I\leq -log(\frac{1}{L})$$

(47) $$Id_{max}=-log(\frac{1}{L})M$$

(48) $$I_{actual}\leq \sum_{i=0}^{\infty }-log(\frac{1}{L_{i}})M{AS}^{i}=-log(\frac{1}{L})M\sum_{i=0}^{\infty }{AS}^{i}$$

(49) $$Bd_{c}=10log\left(\frac{I_{actual}}{Id_{max}}\right)$$

where *L* indicates the total signaling type in order to describe the cost of a single message on the control panel.

By the way, since in this study we tried to design a way that can marginalize the decision-making process to reduce the overhead on the core network controller, which may challenge the computing power of the mobile station, we also conducted experiments on the CPU computing load in experiments and simulations. Table 5 shows the CPU and cache usage rate during the simulation loop. The results show that the average mobile device is fully capable of running the model generated in our proposed way, which is lower than what many other edge computing-related studies require for mobile devices [46,47,48,49,50].

## 7. Discussions and Conclusions

The proposed integrated space–ground communication system architecture represents a significant advancement in addressing the challenges of future communication networks, particularly in managing user surge and reducing network overhead. By combining terrestrial cellular networks, drone networks and satellite networks with slicing technology, our architecture offers a versatile solution that enhances network efficiency and reliability.

This study introduces a novel marginal MS-assisted network resource allocation architecture. Leveraging cellular network slicing technology, it dynamically manages user demands and network conditions. Integration of the DP-DQN model enhances real-time resource allocation decisions for MS, optimizing performance. Furthermore, our proposed feedback method ensures the accuracy and adaptability of the marginalization model, crucial for maintaining optimal network performance in dynamic environments. Through extensive simulations, our DP-DQN-based edge decision method demonstrates superior performance in reducing core network overhead and improving access success rates compared to conventional approaches.

As for the analysis of the DP-DQN model in the numerical results section, we found that taking the location information of mobile devices into account significantly improved the resource utilization of each network. Also, in terms of network slicing, including different physical channels into the type of network slices also helps to improve the degree of integration of the system. At the same time, using the new distributed MS-assisted resource decision model, the system overhead (especially the control panel) is greatly reduced, which gives us reason to believe that in the future communication system, compared with the traditional centralized control, distributed decision-making will have a better prospect.

This study identifies promising future research directions for space–ground integrated systems. Our architecture’s scalability and adaptability extend its applicability beyond traditional networks to smart cities, disaster response, and remote sensing. As demand for seamless connectivity grows, our approach lays a robust foundation for efficient and resilient communication infrastructures. This research advances communication systems by innovatively addressing network resource allocation and decision-making, bridging terrestrial and satellite networks. Our next steps focus on enhancing system integration through improved methods for acquiring and utilizing MS navigation and remote sensing data.

## Figures and Tables

**Table 1:** Summary of parameters in the three training processes.

Training Process	Parameter Descriptions
Round 1	Total Service Types
	Average Data Volume
	Network Congestion
Round 2	Network Side Transmission Power
	Average Delay of the Network
	User Amount in the Coverage
Round 3	Average Data Amount
	MS Location Information
	States of Three Networks
	Records of Past Resource Decisions

**Table 2:** Summary of parameters.

Parameters	Description
R	Reference transmission rate matrix of services
Z	Package size matrix of users
T	Service type matrix of users
$S_{p}$	Priority score matrix of users
$R^{\prime }$	Actual allocated data rate matrix
$P_{kb}\left(t\right)$	Transmitting power matrix of users
*M*	Number of MS per unit time
$B_{kb}$	Allocated bandwidth of a certain user
$P_{kb}\left(t\right)$	Transmitting power of a certain user
$h_{kb}\left(t\right)$	Channel state array of a certain physical link
$\sigma_{k}^{2}\left(t\right)$	Noise power of a certain channel
$L_{i}$	Location parameter of a certain user
$D_{ij}$	Distance between two access node in Km
ρ(t)	MS density of a certain location area
*C*	Cost function of an allocation decision
$C_{ch}$	Capacity of a certain slice
A(t)	Slice allocation states(allocated or not)
Ø	Resource availability of a certain slice
S(t)	User real time parameter (e.x. data amount)
PW(t)	Penalty function of DP-DQN
DA	Congestion Rate of slices
$L_{c}$	The distance from MS to cellular access node
$L_{u}$	The distance from MS to UAV access node
AS	The successful access ratio of several MS
$Bd_{c}$	The network overhead of control panel
$Bd_{u}$	The network overhead of user panel

**Table 3:** Summary of simulation parameter settings.

Parameter	Description	Value
M	Total MS amount.	3000
$M_{U}$	Total MS uplink request amount.	1500
$M_{D}$	Total MS downlink request amount.	1500
$R_{n}$	Total link type amount.	3
$T_{n}$	Total service type amount.	3
$S_{m}$	Minimum data rate of one RB.	3 MBps
${\mathrm{RB}}_{m}$	Maximum RB amount of one slice.	1600
$U_{m}$	Maximum data amount of one request.	2 GBps
$\lambda_{1}$	SF of MS resources request.	alterable
$\lambda_{2}$	SF of MS transmitting power.	alterable
$\lambda_{3}$	SF of MS distance to base station.	alterable
$\lambda_{4}$	SF of MS distance to UAV devices.	alterable
$\lambda_{poission}$	Minimum data rate of one RB.	alterable
${\mathrm{SM}}_{T}$	Total simulation span.	1 hour

**Table 4:** Summary of SF setting in Figure 8.

Parameter	Value (a)	Value (b)	Value (c)	Value (d)
$\lambda_{1}$	0.50	0.50	0.70	0.50
$\lambda_{2}$	0.50	0.50	0.80	0.80
$\lambda_{3}$	0.50	0.80	−0.80	−0.60
$\lambda_{4}$	0.50	0.50	0.50	−0.30

**Table 5:** Summary of CPU parameters during simulation.

Parameter	Average Value
CPU utilization	0.62
CPU base speed	2.80 GHz
CPU speed	3.62 GHz
L1 D-cache	320 KB
L2 D-cache	5.0 MB
L3 D-cache	12.0 MB

## Data Availability

Data are contained within the article.
